# Cardiopulmonary resuscitation during the COVID-19 pandemic: a scientific statement on CPR management protocol of Kasr Al-Ainy University Hospital is presented

**DOI:** 10.1186/s43044-020-00106-9

**Published:** 2020-10-27

**Authors:** Hesham S. Taha, Mirna M. Shaker, Mohamed M. Abdelghany

**Affiliations:** Department of Cardiology, Faculty of Medicine, Cairo University, 27 Nafezet Sheem El Shafae St Kasr Al Ainy, Cairo, 11562 Egypt

**Keywords:** Cardiopulmonary resuscitation, Cardiac arrest, COVID-19

## Abstract

**Background:**

The COVID-19 pandemic poses a major burden to the healthcare system in Egypt, and in the face of a highly infective disease which can prove fatal, healthcare systems need to change their management protocols to meet these new challenges.

**Main body:**

This scientific statement, developed by the cardiology department at Cairo University, emphasized 6 different aspects that are intended to guide healthcare providers during cardiopulmonary resuscitation (CPR) in the era of the COVID-19 pandemic. It highlighted the importance of dealing with all cardiac arrest victims, during the pandemic, as potential COVID-19 cases, and the use of appropriate personal protective equipment (PPE) by health care providers during the procedure. It also stated that the CPR procedure should be done in a separate room with the door closed and that the number of providers present during the procedure should be limited to only those who are essential for patient resuscitation. It also stressed that family members and accompanying personnel of patients with possible COVID-19 should not be in the vicinity of CPR site. The statement also pointed out that CPR procedure should be done in the standard manner with precautions to minimize spread of infection to the staff and accompanying people. Early intubation was prioritized, and the use of rapid sequence intubation with appropriate PPE was recommended. For delivery of CPR for the prone ventilated patient, delivery of chest compressions by pressing the patient’s back, while a team prepares to turn the patient supine, was recommended. During intra-hospital transport, it was emphasized that the receiving intensive care unit (ICU) should be notified about the possibility of the patient being COVID-19 positive, so that appropriate infection control precautions are taken.

**Conclusion:**

Cardiopulmonary resuscitation of cardiac arrest patients in the COVID-19 era poses a significant challenge, and all health care providers should deal with any cardiac arrest victim presenting to the emergency department as potential COVID-19 suspects and should use the appropriate PPE.

## Background

Prior to the COVID-19 pandemic, survival from cardiac arrest (CA) had steadily improved in the cardiology department, at Faculty of Medicine, Cairo University, thanks to the continuous cardiopulmonary resuscitation (CPR) training and quality improvement measures including auditing CPR outcomes as well as morbidity and mortality (M & M) meetings [1].

Now facing the pandemic, up to 6% of COVID-19 positive patients become critically ill and may develop CA [2–4]. The underlying cause of CA in patients with severe COVID-19 pneumonia was mainly respiratory (87.5%) [5], in a report from Wuhan, China, with the underlying rhythm asystole in the majority of cases (89.7%). Different factors predispose these patients to CA including hypoxemic respiratory failure, myocardial injury, ventricular arrhythmias, and shock [6–8].

As CPR stands out as an inherently risky activity that involves aerosol-generating steps [9], rescuers must continuously balance the immediate needs of the patients with their own safety.

Cardiologists, being front-liners, and usually taking the lead in CPR activity, had to have a scientific statement on how to deal with cardiac arrest during the COVID-19 pandemic.

The statement contained herein is based on international guidelines [10, 11] as well as local expert opinion. As countries are at different stages of the pandemic, we do realize that there may be some variation in practice in different regions.

## Principle CPR recommendations during the COVID-19 pandemic

### Pre-hospital/emergency department

1. During cardiac arrest, first responders should don an N95 (or best available) mask.

2. One rescuer should confirm pulselessness and non-responsiveness of the victim and start compression only CPR (rescue breaths may be needed in children).

3. He (or a second rescuer) must inquire about the possibility of COVID-19 infection of the patient, and the hospital emergency department (ED) should be notified in advance that they may be receiving a patient with possible COVID-19 infection. The query process should never delay any immediate lifesaving intervention.

4. If automated external defibrillation (AED) is available, it should be used early as indicated.

5. If the dispatcher did not provide information about the possible COVID-19 status of the patient, emergency medical services (EMS) clinicians should take appropriate precautions when responding to any patient especially with manifestations of a respiratory infection.

### In-hospital

1. CPR should (when possible) be done in a single-person room with the door closed.

2. Negative pressure rooms are an advantage, but rarely present in the ED.

3. The room should be disinfected following the procedure.

### Personnel

1. The number of providers present during the procedure should be limited to only those necessary for resuscitation.

### Personal protective equipment

1. Respiratory protection: put on a respirator or surgical facemask (if a respirator is not available) before dealing with the patient. N95 respirators or others that offer a higher level of protection should be used instead of a facemask when performing an aerosol-generating procedure (e.g., CPR, endotracheal intubation, non-invasive ventilation).

2. Eye protection

3. Gloves

4. Gowns

### Procedures

Cardiopulmonary resuscitation is done in the standard manner, with the following precautions to minimize spread of infection to the staff and accompanying people:

1. When assessing breathing, look for normal breathing but do not place your face next to the victims’ mouth and nose.

2. It is preferable to pause chest compressions during ventilations to minimize the risk of spread of infection.

3. Lay rescuers are to consider compression-only CPR, but may accept to deliver rescue breaths to children in addition to chest compressions.

4. If available, it is advisable to use adhesive pads for defibrillation, hence delivering shock without direct contact between the defibrillator operator and the patient.

5. Intubation and mechanical ventilation:

• If intubation is needed, rapid sequence intubation, with appropriate personal protective equipment (PPE), may be used.

• Proceeding directly to endotracheal intubation in patients with acute respiratory failure may be considered.

• Avoid, as possible, procedures which generate aerosols (e.g., non-invasive positive pressure ventilation, nebulizers).

• Ventilatory equipment should be provided with HEPA filtration for expired air.

6. As COVID-19 patients are occasionally managed in the prone position to improve oxygenation, it is advisable that:

• In the event of cardiac arrest in the un-intubated, prone patient, while wearing the correct PPE, the rescuer should turn the patient supine before starting chest compressions.

• In the event of cardiac arrest in an intubated patient who is prone, it is possible to deliver chest compressions by pressing the patient’s back (between the scapulae at the usual depth and rate of 5 to 6 cm at a rate of 2 compressions per second) while a team prepares to turn the patient supine.

• Defibrillator pads may be placed with patient in the prone position either anterior-posterior or bi-axillary.

7. Family members and other contacts of patients with possible COVID-19 should not be in the vicinity of CPR site while CPR is ongoing.

### Intra-hospital transport

1. EMS personnel should notify the receiving intensive care unit (ICU) if the patient has an exposure history and/or manifestations suggestive of COVID-19.

2. The hospital path (including elevators) should be cleared of visitors before transfer.

3. Transporting personnel should be donning full PPE.

4. Avoid crowded areas and crowded times as possible.

5. Keep the patient with suspected COVID-19 separated from other people as much as possible.

**Fig. 1:**
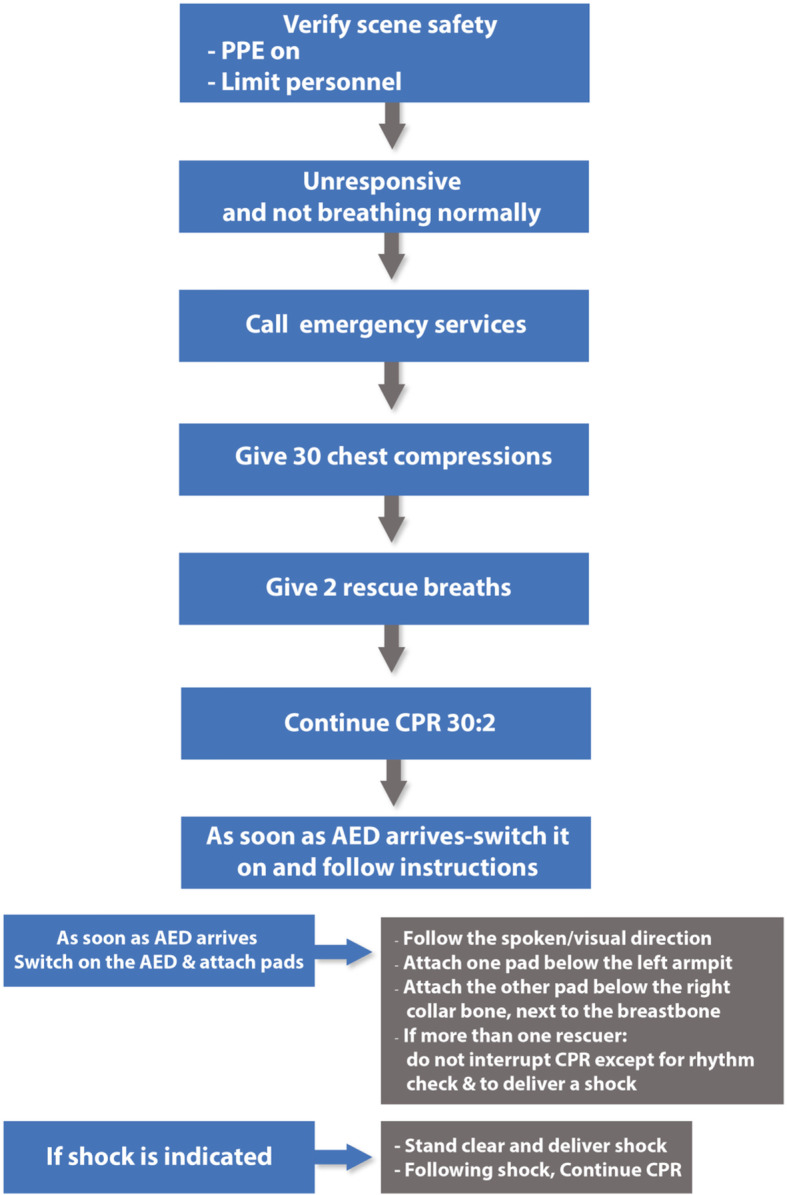
Basic life support algorithm for adults during the COVID-19 pandemic

**Fig. 2:**
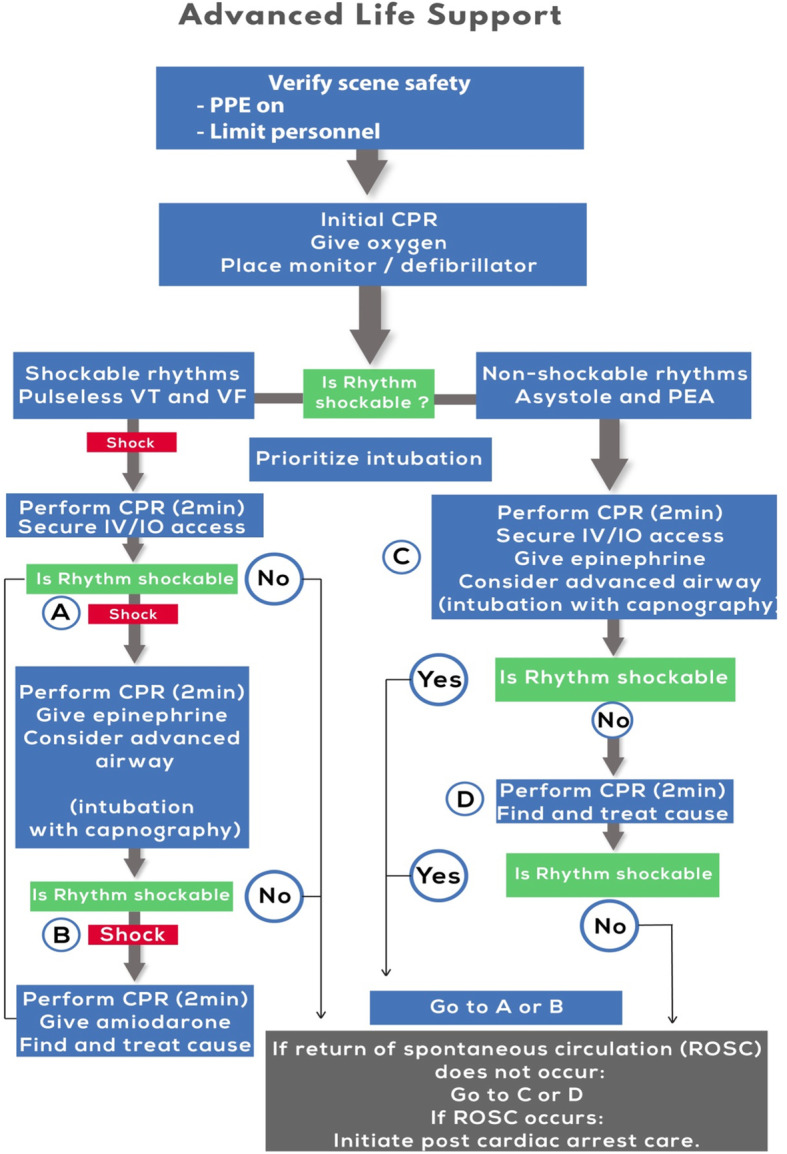
Advanced cardiac life support algorithm for adults during the COVID-19 pandemic

Figures 1 and 2 illustrate the modified basic and advanced adult cardiac life support algorithm during the COVID-19 pandemic.

## Conclusions

The challenge in the development of the CPR scientific statement was to ensure that patients with or without COVID-19 who experience CA get the best possible chance of survival without compromising the safety of rescuers. The statement emphasized that during the pandemic, all staff should deal with any cardiac arrest victim presenting to the emergency department as a potential COVID-19 suspect and should use the appropriate PPE. CPR procedure should be done in a single-person room with the door closed if possible, and the least number of necessary health care providers present. CPR is done in the standard manner, with precautions to minimize spread of infection and some modifications as prioritizing early intubation, and resuscitating intubated patient who are in prone position by pressing the patient’s back to deliver chest compressions while a team prepares to turn the patient supine.

## Data Availability

“Not applicable”

## Abbreviations

AED: Automated external defibrillation
CA: Cardiac arrest
CPR: Cardiopulmonary resuscitation
COVID-19: Coronavirus disease 2019
ED: Emergency department
EMS: Emergency medical services
ICU: Intensive care unit
M & M: Morbidity and mortality
PPE: Personal protective equipment
